# Five-year analysis of clinical presentations and predictors of stroke mortality in rural Southwestern Nigeria: A retrospective observational study

**DOI:** 10.1016/j.afjem.2021.10.005

**Published:** 2021-12-28

**Authors:** Azeez Oyemomi Ibrahim, Olabode Muftau Shabi, Tosin Anthony Agbesanwa, Paul Olowoyo

**Affiliations:** a Department of Family medicine, Federal Teaching Hospital, Ido-Ekiti, Ekiti State, Nigeria; b Department of Family Medicine, Afe Babalola University, Ado-Ekiti, Ekiti State, Nigeria; c Department of Family Medicine, Ekiti State University Teaching Hospital, Ado-Ekiti, Nigeria; d Department of Medicine, Afe Babalola University, Ado-Ekiti College of Medicine and Health Sciences, Ekiti State, Nigeria

**Keywords:** AED, Accident and Emergency Department, FETHI, Federal teaching hospital, Ido-Ekiti, IS, Ischemic Stroke, HS, Haemorrhagic Stroke, GCS, Glascow Coma Score, SBP, Systolic blood pressure, DBP, Diastolic blood pressure, BMI, Body Mass Index, AF, Atrial fibrillation, RR, Respiratory rate, HR, Heart rate, SSA, Sub- Saharan Africa, Stroke, Predictors, Mortality, Rural, Nigeria

## Abstract

**Introduction:**

Stroke mortality and its predictors are important outcome measures in stroke epidemiological studies and clinical trials. There is an observed paucity of data regarding the clinical presentations and predictors of stroke mortality in Southwestern Nigeria. Few available related studies have centred on hospitals in the urban and sub-urban areas; however, none in the rural settings. This study, therefore, focuses on the clinical presentations and predictors of stroke mortality at the adult Emergency Centre of a tertiary hospital situated in rural Southwestern Nigeria.

**Methods:**

A retrospective survey, using data form and standardized questionnaire, was used to study the patients admitted for stroke between January 2015 and December 2019. The data were analysed using SPSS Version 22.0. The results were presented in descriptive and tabular formats.

**Results:**

A total of 276 patients were studied. Their mean age was 67.3 ± 11.1 years. The most common clinical presentations were hemiparesis and cranial nerve deficit. The case of fatality was 10.1%. The predictors of stroke mortality were age ≥65 years [(AOR = 12.752; 95% CI: (1.022–159.190), *p* = 0.048)], Glascow coma score <8 [(AOR = 50.348; 95% CI: (7.779–325.866), *p* < 0.001)], uncontrolled blood pressure [(AOR = 23.321; 95% CI: (2.449–221.927), *p* = 0.006)], presence of atrial fibrillation [(AOR = 16.456; 95% CI: (2.169–169.336), *p* = 0.009)], convulsion [(AOR = 25.889; 95% CI: (2.374–282.296), *p* = 0.008)], heart failure [(AOR = 30.284; 95% CI: (3.265–256.347), *p* < 0.001)], and a repeat stroke [(AOR = 32.617; 95% CI: (2.410–441.381), p = 0.009)].

**Conclusion:**

The 7-day fatality was 10.1%. The predictors of stroke mortality were poor Glascow coma score, uncontrolled blood pressure, atrial fibrillation, heart failure, convulsion and a repeat stroke. This study strengthens the argument on the higher prevalence of stroke and its mortality in rural Southwestern Nigeria. Our findings may provide an impetus for prospective research on this outcome.

## African relevance

• Majority of people in sub-Saharan African reside in rural areas.
• Many hospitals in rural and sub-urban centres do not have adequate personnel and infrastructures.
• There are limited data on clinical presentations and predictors of stroke mortality; and are largely from urban and semi-urban health care facilities
• The results of the present study would guide the stakeholders on how to reduce stroke mortality in rural centres

## Introduction

Stroke is defined as a progressing and a clinical event which presents as focal or global neurological deficit, with symptoms lasting more than 24 h or leading to death, with no apparent cause other than vascular origin 1., 2.. Stroke is one of the causes of morbidity and mortality worldwide, affecting both male and female subjects. Research into this challenge has been the concern of many countries 1., 2..

According to the World Health Organization (WHO), stroke accounts for approximately 5.8 million deaths per year globally 2., [3]. In Africa, stroke has been found to account for 0.9–4.0% of hospital admissions and 2.8% - 4.5% of total deaths [4]. In Nigeria, stroke is responsible for 4–17% of deaths 5., 6.. Consequently, the disease is now being recognized as a leading cause of death in Sub-Saharan Africa (SSA) [2]. This negative impact will likely increase in the future due to the ongoing demographic and lifestyle changes observed in low and middle-income countries (LMICs) [7].

Previous studies have identified stroke subtype, patients' demography (such as age and gender), physiological admission parameters (such as blood pressure, blood glucose, level of consciousness, disease severity, and the presence of complications) as contributing predictors of stroke mortality worldwide 4., 8.. Haemorrhagic stroke (HS) is found to account for the highest risk of early death, from cerebral oedema expansion, hematoma, or cardiac complications [5]. Repeat stroke, frequent seizures and immobilization have also been established to be common causes of late death [9]. Patients with stroke have been observed to get better when thrombolytic therapy is administered within the first 3 h after the onset of ischemic stroke (IS). However, there is an observed paucity of data regarding the clinical presentations and predictors of stroke mortality in Southwestern Nigeria. Few available related studies were conducted in health facilities located in the urban centres, and none has been done in the health facilities located in the rural areas where the majority of southwestern Nigeria populace resides. Therefore, the management of stroke in rural areas is the basis of this study. Data on stroke mortality in rural settings are critical to enhancing the health system's preparedness towards meeting patient expectations, leading to improved health care delivery and outcomes 7., 10.. This study aims at determining the clinical presentations and predictors of stroke mortality at the adult Emergency Centre of a tertiary hospital located in a rural setting in Southwestern Nigeria.

## Methods

### Study area

The study was carried out at the adult Emergency Centre of a tertiary hospital situated in a rural community which was about 15 km from the (Ekiti) State capital. The total landmass of the community is 332km^2^, with a projected population of 225,305 [11]. The majority of the populations are employed within the informal sector mainly as farmers and traders. Also, a sizeable population were senior citizens who have retired from the formal employment sector. The study centre has 180 beds and serves as a referral centre to patients from private and government-owned health facilities in its environs. The hospital offers emergency services to medical and surgical patients admitted, most of the time, without prior appointment. There are fourteen beds for male patients and ten for females in the hospital. The medical team usually includes the consultant physician specialists, medical officers, internists and supported by other healthcare workers. Nevertheless, the consultant neurologist oversees the management of patients with stroke.

### Study design

This was a descriptive, retrospective review of hospital records of patients with stroke that were admitted at the adult AED between 1st January 2015 and 31st December 2019.

Study population: This included all patients with stroke who were registered and admitted between 2015 and 2019.

### Sample size

A total of 276 patients with stroke whose medical records were complete, and diagnoses confirmed by the Computed Tomography (CT) imaging were purposively selected for this study. This was derived from the total number of 341 patients admitted for stroke. Among these admissions, three hundred and nine (309) were confirmed by Computed Tomography (CT) imaging to have suffered as stroke. Of these admissions, only 276 patients who had complete medical records were used for this study.

### Inclusion criteria

Patients with stroke who were confirmed by imaging (CT scan) and had complete data.

### Exclusion criteria

Patients with stroke whose data were incomplete, not available or whose initial diagnosis of stroke was later changed to other cause.

### Instruments for data collection

The instruments for data collection were designed and developed by the researchers. The instruments included a data form and a standardized questionnaire, which contained the variables to be measured based on the previous literature from the WHO approach to stroke surveillance [12].

### Methods of data collection

The data form and the standardized questionnaire were used to obtain information from the case records of each patient on admission or discharged in the nursing report books. Information retrieved included the date and year of admission, demographic profile, presenting features, type of stroke, blood pressure on admission, heart rate (HR), body core temperature and random plasma glucose. Other information included the Glascow coma score (GCS), the time interval between the onset of stroke and hospital arrival, and the presence of seizure on admission. The electrocardiogram (ECG) report for each patient was examined for evidence of cardiac diseases like atrial fibrillation (AF) and heart failure. In case of an eventual death, the time interval between the onset of stroke admission and death was also extracted and recorded. Clinical outcomes were also considered. The retrieval of clinical outcomes was to show if the patient was discharged home, Discharged Against Medical Advice (DAMA), referred to another facility or died. The data were collected by two trained casualty officers, a nursing officer, and supported by one resident doctor from the neurology unit. The data were cross-checked by the corresponding author.

### Data entry and analysis

All data collected were checked for completeness and entered into Epi info version 7. These were later exported to SPSS version 22.0 for analysis. Continuous data were analysed as Mean standard deviation, while categorical data were analysed and presented as frequencies and percentages. Variables of interest presented as categorical data were compared using Pearson's chi-square analysis and *p* < 0.05 was considered statistically significant. Multivariate logistic regression model was used to determine the predictors of stroke mortality.

### Ethical clearance and consideration

The institution's Ethics and Research Committee (ERC) approved the study (ERC/2020/08/25/402A). Also, information obtained from each patient was kept anonymous and confidential.

## Results

**Fig. 1:**
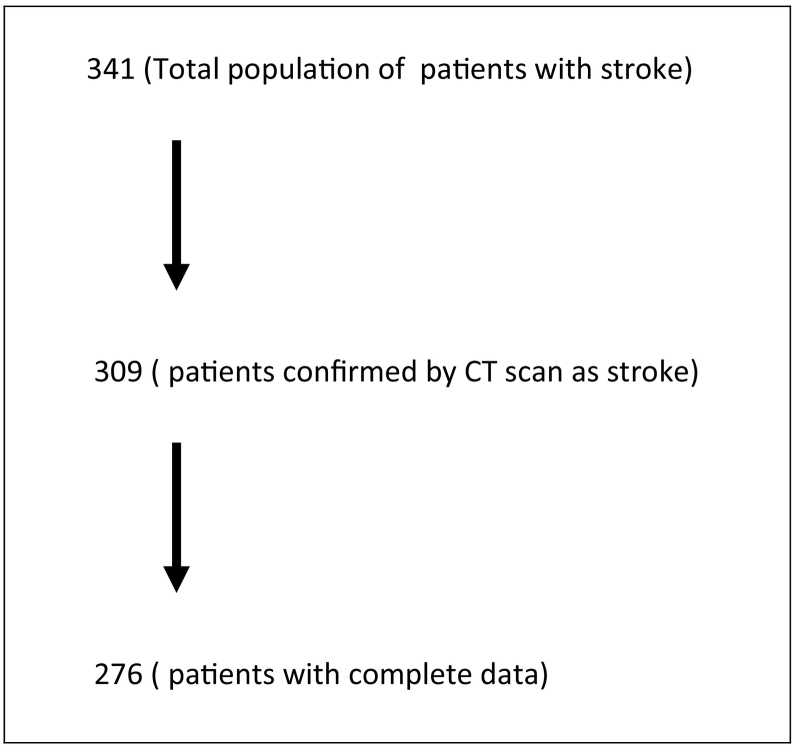
Flow chart of patients with stroke.

Between January 2015 and December 2019, there were 5944 admissions at the adult Emergency Centre of the study institution. Among these admissions were 341 (5.7%) patients, who were admitted for stroke. Of these admissions, 309 patients were confirmed by CT imaging to have suffered a stroke. Again, out of these, only 276 (89.3%) patients had complete medical records and were used for this study. The medical records of the remaining 33 (10.7%) patients were incomplete and therefore were not included in analysis, as seen in Fig. 1.

The mean age of the studied patients was 67.3 ± 11.1 years, and the majority, 161 (58.3%), were 65 years and above. There were more males patients, with a frequency of 167 (60.5%), as seen in Table 1.

**Table 1:** Age and sex distribution in stroke subtypes (*N* = 276).

Variable	Type of stroke				
	Ischemic n (%) *N* = 166	Haemorrhagic n (%) *N* = 110	Total N (%) N = 276	Chi square	*p*-value
Age (in years)					
40–64	70 (60.9)	45 (39.1)	115 (41.7)	0.043	0.835
≥ 65	96 (59.6)	65 (40.4)	161 (58.3)		
*Mean age ± SD*	*67*.*9 ± 13*.*8*	*66*.*4 ± 6*.*5*	*67*.*3 ± 11*.*1*	*1*.*126*^*t*^	*0*.*261*

Sex					
Male	106 (63.5)	61 (36.5)	167 (60.5)	1.954	0.162
Female	60 (55.5)	49 (45.0)	109 (39.5)		

t – Independent *t*-test.

The majority of the patients, 226 (82.0%) and 166 (60.0%) presented with hemiparesis(plegia) and cranial nerve deficit, respectively. Besides, most of the patients who suffered Ischemic stroke (IS) presented with headache (47.6) and aphasia (26.5%). Also, most of the patients who suffered haemorrhagic stroke (HS) presented with headaches (48.2%) and altered sensorium (47.3%). There was a statistically significant difference between the stroke subtype in the studied patients and altered sensorium (*p* < 0.001) and vomiting (p < 0.001), as seen in Table 2.

**Table 2:** Stroke clinical presentation in the studied patients (*N* = 276).

Variable	Stroke subtype				
	Ischemic n (%) *N* = 166	Haemorrhagic n (%) *N* = 110	Total N (%) N = 276	Chi square	*p*-value
Stroke clinical presentation					
Hemiparesis (plegia)	140 (84.3)	86 (78.2)	226 (82.0)	1.690	0.194
Cranial nerve deficit	105 (63.3)	61 (55.5)	166 (60.0)	1.679	0.195
Headache	79 (47.6)	53 (48.2)	132 (48.0)	0.009	0.923
Altered Sensorium	40 (24.1)	52 (47.3)	92 (33.3)	15.992	**<0**.**001**
Aphasia	44 (26.5)	34 (30.9)	78 (28.3)	0.634	0.426
Vomiting	24 (14.5)	38 (34.5)	62 (22.5)	15.327	**<0**.**001**
Slurred Speech	30 (18.1)	22 (20.0)	52 (18.8)	0.161	0.688
Dizziness	18 (10.8)	12 (10.9)	30 (10.8)	0.000	0.986
Urinary incontinence	14 (8.4)	8 (7.3)	22 (8.0)	0.122	0.727
Convulsion	10 (6.0)	4 (3.6)	14 (5.2)	0.783	0.376

The majority of the patients, 102 (37.0%), arrived at the hospital between 4.5 and 12 h from the onset of stroke. The majority of patients who suffered HS, 72 (65.5%), had arrived at the hospital earlier than those patients who suffered IS, 30 (18.1%). There were 111 (40.2%) patients with GCS score ≤ 8. The mean GCS score was 7.9 ± 4.2 and was poor for patients who suffered HS (*p* < 0.001). Irrespective of their stroke subtypes, the majority of patients, 181 (65.6%), had uncontrolled systolic blood pressure (SBP) (≥140 mmHg) and 219 (79.3%), had uncontrolled diastolic blood pressure (DBP) (≥90 mmHg). The majority, 249 (90.2%), had stroke for the first time. There was a presence of atrial fibrillation on ECG in 52 (18.8%) of patients who suffered a stroke (*p* = 0.009). In all, there was a statistically significant association between the stroke subtypes and the time interval from the onset of stroke to hospital arrival (*p* < 0.001), GCS scores (p < 0.001), RR (p < 0.001), HR (*p* = 0.019), SBP (p < 0.001), DBP (p < 0.001), and atrial fibrillation (p = 0.009), Table 3.

**Table 3:** Admission stroke event factors and clinical findings in the studied patients (*N* = 276).

Variable	Type of stroke				
	Ischemic n (%) N = 166	Haemorrhagic n (%) *N* = 110	Total N (%) N = 276	Chi square	p
Time interval from onset of stroke to hospital arrival (hours)					
≤ 4.5	3 (1.8)	18 (16.4)	21 (7.6)	117.815	**<0**.**001**
4.51–12.00	30 (18.1)	72 (65.5)	102 (37.0)		
12.01–24.00	22 (13.3)	14 (12.7)	36 (13.0)		
24.01–72.00	45 (27.1)	4 (3.6)	49 (17.8)		
> 72.00	66 (39.8)	2 (1.8)	68 (24.6)		
*Median* (*IQR*)	*48*.*0* (*150*.*0*)	*8*.*0* (*6*.*0*)	*18*.*0* (*54*.*0*)	*2141*.*000*^*MWU*^	***<0***.***001***
Glascow coma score					
Poor GCS (≤ 8)	40 (24.1)	71 (64.5)	111 (40.2)	46.932	**<0**.**001**
Moderate GCS (9–12)	50 (30.1)	10 (9.1)	60 (21.7)		
Good GCS (13–15)	76 (45.8)	29 (26.4)	105 (38 0.0)		
*Mean ± SD*	*11*.*2 ± 3*.*4*	*7*.*9 ± 4*.*2*	*9*.*9 ± 4*.*1*	*7*.*293*^*t*^	***<0***.***001***
Respiratory rate (cycle/min)					
12–20	98 (59.0)	8 (7.3)	106 (38.4)	74.938	**<0**.**001**
> 20	68 (41.0)	102 (92.7)	170 (61.6)		
*Mean ± SD*	*22*.*0 ± 4*.*1*	*24*.*8 ± 2*.*5*	*23*.*1 ± 3*.*8*	*−6*.*413*^*t*^	***<0***.***001***
Heart rate					
Normal (60–100 bpm)	146 (88.0)	85 (77.3)	231 (83.7)	5.529	**0**.**019**
Abnormal (> 100 bpm)	20 (12.0)	25 (22.7)	45 (16.3)		
*Mean ± SD*	*83*.*9 ± 12*.*3*	*89*.*7 ± 12*.*9*	*86*.*2 ± 12*.*8*	*−3*.*701*^*t*^	***<0***.***001***
Systolic BP (mmHg)					
90–120	60 (36.1)	14 (12.7)	74 (26.8)	32.748	**<0**.**001**
121–139	3 (1.8)	18 (16.4)	21 (7.6)		
≥ 140	103 (62.0)	78 (70.9)	181 (65.6)		
*Mean ± SD*	*150*.*4 ±* *28*.*7*	*157*.*6 ± 26*.*1*	*153*.*3 ± 27*.*9*	*−2*.*056*^*t*^	***0***.***041***
Diastolic BP (mmHg)					
60–80	45 (27.1)	3 (2.7)	48 (17.4)	32.389	**<0**.**001**
81–89	8 (4.8)	1 (0.9)	9 (3.3)		
≥ 90	113 (68.1)	106 (96.4)	219 (79.3)		
*Mean ± SD*	*94*.*4 ± 15*.*2*	*103*.*2 ± 7*.*4*	*97*.*9 ± 13*.*4*	*−5*.*678*^*t*^	***<0***.***001***
Temperature (°C)					
Normal (36.0–37.2)	140 (84.3)	83 (75.5)	223 (80.8)	3.365	0.067
Hyperthermia (> 37.2)	26 (15.7)	27 (24.5)	53 (19.2)		
*Mean ± SD*	*37*.*0 ± 0*.*7*	*37*.*8 ± 4*.*7*	*37*.*3 ± 3*.*0*	*−2*.*071*	***0***.***039***
Number of episodes					
First ever	148 (89.2)	101 (91.8)	249 (90.2)	0.531	0.466
Recurrent	18 (10.8)	9 (8.2)	27 (9.8)		
Body mass index (Kg/m^2^)					
Normal weight (18.5–24.9)	102 (61.4)	80 (72.7)	182 (65.9)	3.986	0.136
Overweight (25.0–29.9)	50 (30.1)	22 (20.0)	72 (26.1)	3.986	0.136
Obese (≥ 30)	14 (8.4)	8 (7.3)	22 (8.0)		
*Mean ± SD*	*24*.*3 ± 3*.*9*	*24*.*1 ± 3*.*6*	*24*.*2 ± 3*.*8*	*0*.*445*^*t*^	*0*.*657*
RPG Glucose (mmol/L)					
Normal (7.0–11.0)	140 (84.3)	97 (88.2)	237 (85.9)	0.806	0.369
High (>11.0)	26 (15.7)	13 (11.8)	39 (11.8)		
*Mean ± SD*	*10*.*2 ± 3*.*0*	*10*.*1 ± 2*.*4*	*10*.*2 ± 2*.*8*	*0*.*632*^*t*^	*0*.*718*
Atrial fibrillation on ECG (AF)	23 (13.9)	29 (26.4)	52 (18.8)	6.769	**0**.**009**
Convulsion on Admission (COA)	10 (6.0)	6 (5.5)	16 (5.8)	0.039	0.843

MWU – Mann-Whitney *U* test – independent t-test

The clinical outcomes of the studied patients showed that the majority, 196 (71.0%), were discharged home, while 46 (16.7%) were DAMA, and 28 (10.1%) were dead. There was a statistically significant difference in the clinical outcomes of the studied patients (*p* < 0.001), Table 4.

**Table 4:** Clinical outcomes of the studied patients (*N* = 276).

Variable	Type of stroke				
	Ischemic n (%) N = 166	Haemorrhagic n (%) *N* = 110	Total n (%) N = 276	Chi square	p-value
				37.432	**<0**.**001**
Discharged home	126 (75.9)	70 (63.3)	196 (71.0)		
DAMA	28 (16.9)	18 (16.4)	46 (16.7)		
Dead	8 (4.8)	20 (18.2)	28 (10.1)		
Referred to other facility	4 (2.4)	2 (1.8)	6 (2.2)		
**Total**	166 (100.0)	110 (100.0)	276 (100.0)		

In all, 28 (10.1%) patients who suffered a stroke died within one week of admission. The majority of deaths, 17 (60.7%), were males when compared to females who had strokes as at the time of the study. Also, 24 (85.7%) of fatalities occurred among the elderly patients (≥65 years). The cumulative case of fatality rates among the patients with stroke at AED were 2.9% within the first 24 h, 7.2% at 72 h and 10.1% at one week. There was a statistically significant difference between the stroke subtypes and pattern of stroke mortality by genders (*p* = 0.001), Table 5.

**Table 5:** Pattern of stroke mortality in the studied patients (*N* = 28).

Variable	Type of stroke				
	Ischemic n (%) N = 11	Haemorrhagic n (%) *N* = 17	Total N (%) N = 28	Chi square	*p*-value
Age (in years)					
40–64	2 (18.2)	2 (11.8)	4 (14.3)	0.225	0.636
≥ 65	9 (81.8)	15 (88.2)	24 (85.7)		
Sex					
Male	11 (100.0)	6 (35.3)	17 (60.7)	11.723	**0**.**001**
Female	0 (0.0)	11 (64.7)	11 (39.3)		
Period of death from the onset of admission					
< 24 h	2 (18.2)	6 (35.3)	8 (28.6)	1.098	0.578
24 h – 72 h	5 (45.5)	7 (41.2)	12 (42.9)		
72 h – 1 week	4 (36.4)	4 (23.5)	8 (28.6)		

Univariate analysis and multivariate logistic regression were conducted to analyse the predictors of emergency centre mortality. Using the univariate analysis, age ≥65 years [COR = 4.861; 95% CI: (1.638–14.426), *p* = 0.002], GCS <8 [COR = 12.685; 95% CI: (4.633–37.733), *p* = 0.001], high B.P [COR = 4.073; 95% CI: (1.758–9.437), *p* < 0.001], presence of atrial fibrillation [COR =5.526; 95% CI: (2.40–12.515), *p* < 0.001], convulsion [COR =22.407; 95% CI: (7.313–68.653), p < 0.001], heart failure [COR =5.200; 95% CI: (2.303–11.741), p < 0.001], repeat stroke [COR = 14.486; 95% CI: (5.785–36.270), p < 0.001], and HS [COR = 2.576; 95% CI: (1.156–5.737), *p* = 0.017], were associated with stroke mortality.

Subsequent multivariate logistic regression model showed that; age ≥ 65 years [(AOR = 12.752; 95% CI: (1.022–159.190), *p* = 0.048)], GCS scores <8 [(AOR = 50.348; 95% CI: (7.779–325.866), *p* < 0.001)], uncontrolled B.P [(AOR = 23.321; 95% CI: (2.449–221.927), *p* = 0.006)], presence of atrial fibrillation [(AOR = 16.456; 95% CI: (2.169–169.336), *p* = 0.009)], convulsion [(AOR = 25.889; 95% CI: (2.374–282.296), *p* = 0.008)], heart failure [(AOR = 30.284; 95% CI: (3.265–256.347), *p* < 0.001)], and a repeat stroke [(AOR = 32.617; 95% CI: (2.410–441.381), p = 0.009)] were the independent predictors of stroke mortality, Table 6.

**Table 6:** Multivariate binary logistic regression for the independent predictors of stroke mortality in the studied patients (*N* = 276).

Variable	Bivariate analysis		Multivariate analysis	
	OR (95% CI)	P	OR (95% CI)	P
Age - ≥65	4.861 (1.638–14.426)	**0**.**002**	12.752 (1.022–159.190)	**0**.**048**
Male Sex	1.010 (0.454–2.247)	0.981	0.358 (0.057–2.228)	0.271
GCS <8	12.685 (4.633–37.733)	**<0**.**001**	50.348 (7.779–325.866)	**<0**.**001**
Heart rate (>100)	3.705 (0.853–16.098)	0.062	2.877 (0.388–21.355)	0.301
Temperature >37.2 °C	2.196 (0.932–5.176)	0.067	0.088 (0.008–0.914)	**0**.**042**
Respiratory Rate >20	0.960 (0.431–2.137)	0.920	0.178 (0.024–1.345)	0.094
High B.P (≥140/90)	4.073 (1.758–9.437)	**0**.**001**	23.312 (2.449–221.927)	**0**.**006**
Atrial fibrillation	5.526 (2.40–12.515)	**<0**.**001**	16.456 (2.169–169.336)	**0**.**009**
Presence of Convulsion	22.407 (7.313–68.653)	**<0**.**001**	25.889 (2.374–282.296)	**0**.**008**
Presence of Heart failure	5.200 (2.303–11.741)	**<0**.**001**	30.284 (3.265–256.347)	**<0**.**001**
Repeat stroke	14.486 (5.785–36.270)	**<0**.**001**	32.617 (2.410–441.381)	**0**.**009**
Haemorrhagic stroke	2.576 (1.156–5.737)	**0**.**017**	1.569 (0.171–14.389)	0.690

## Discussion

There is paucity of data on clinical presentations and predictors of stroke mortality in rural Southwestern Nigeria, even though there is a large body of information on the same subject from the western literature. This is primarily a clinical study on stroke which addresses the epidemiology, pattern of presentation and clinical outcomes of patients that suffered stroke, as observed in a tertiary hospital situated in a rural community of Southwestern Nigeria.

The patients with stroke constituted 5.7% of total emergency admissions within the period of the study. This is higher than 0.9–4.0% which has been reported in other parts of Africa 2., 9.. This finding further strengthens the argument on the high prevalence of stroke in rural Southwestern Nigeria. This retrospective survey shows that the mean age of the patients in this study was 67.3 ± 11.1. This is consistent with the findings in other studies, which reported the mean age to be between 50 and 70 years for patients who had stroke 9., 13.. The above age range is where the risk factors for stroke are most prevalent. The higher percentage of stroke in male patients over females was consistent with several other hospital-based studies conducted in Nigeria and other African studies 13., 14.. The absence of vascular protection of endogenous oestrogen in males might be responsible for this observation 14., 15.. Conversely, some other studies have found stroke to be common in females than males 10., 16.. The preponderance of female patients with stroke in the other studies could be due to the high use of contraception and pregnancy-related disorders 10., 16.. However, there is no significant sex predilection to a particular stroke subtype in this study, and Ogbole et al. [17] corroborate this finding.

The majority of our patients showed severe neurological deficits with paraplegia and cranial nerve deficit observed as the major presentations in both stroke subtypes. This is in agreement with the study by Fekadu et al. [18]. We found that the presentation time at the hospital varied by stroke subtype. Patients with HS presented earlier than the patients who had IS, which is consistent with other studies 18., 19.. This could be because some patients with IS present with only mild deficits, and such patients may be hoping that symptoms would resolve spontaneously. With recent advances in thrombolytic therapy for eligible patients with stroke who present within 3 h of ictus, the need for early presentation and imaging diagnosis is paramount to stroke management to achieve a good outcome. None of our patients presented within the time frame of 3 h of ictus. Our median presentation time was 54 h, which was longer than 27 h found by Fekadu et al. [18], and 12.9 h found by Carvalho et al. [20]. The delay may be due to poor access road networks and inadequate transport systems being experienced in the rural settings of Nigeria. Other reasons may be poverty, ignorance, and wrong cultural or religious beliefs, such as attributing the cause of illness to spiritual attacks. Therefore, deliberate measures need to be implemented to address the causes of this delay. Our mean respiratory rate (RR) was 23.1 ± 3.8 (cycle/s) and this was similar to those recorded by Fekadu et al. [18] but higher than those recorded by Gebremariam et al. [21]. The high respiratory rate recorded in this study may be due to the presence of co-morbid ailments found in these patients. Most patients' blood pressure was elevated above the normal range and this was consistent with the findings recorded by other studies 19., [21]. Of the total patients, 9.8% presented with a repeat stroke, and this finding was consistent with the findings by other studies 10., 18. but higher than what was found by Obiako et al. [23]. The difference may be due to stroke awareness variances and poorly controlled vascular risk factors 9., 23..

Of the total patients, less than half exhibited atrial fibrillation on ECG. This is higher than what was found by Russell et al. [9] and could be due to the availability of ECG that was done for all our patients when compared to the findings by other studies 9., 24.. Few patients exhibited convulsion while on admission, and this was similar to the findings by other studies 25., 26.. This may be due to physical exhaustion and sympathetic overactivity [23]. Most patients presented with normal HR, body core temperature, body mass index, and RPG and this is similar to the findings by other studies 14., 19., [21].

The clinical outcomes in this study appear to be good regarding the number of patients who were successfully treated and discharged or referred to other facilities. The availability of CT scan imaging and ECG test for all patients, which may assist in early diagnosis and treatment, may be responsible for these outcomes. However, the high number of patients that were DAMA was a source of concern. Some DAMA patients lacked the funds to pay for the services required. Some patients lost hope, especially when recovery was slow, and preferred to die at home or obtain treatment from the alternative medical practitioners. DAMA, most often, was observed to be due to wrong cultural or religious beliefs, attributing the cause of illness to spiritual attacks. Therefore, effective and sustained health education and communication strategies in the rural setting may be needed to improve early presentation and access to quality health care services. The enrolment of rural dwellers on the National Health Insurance Scheme (NHIS) should also be intensified to reduce the incidence of DAMAs on the grounds of financial constraints in our hospitals.

In this study, the pattern of mortality observed within 24 h, 72 h, and one week agrees with the pattern observed in the aggregated data in WHO's study [27]. The mortality rate observed in this study was higher for patients above the age of 65 years. This agrees with studies in Caucasians where the risk of death in people less than 45 years is low [28]. In our study, there was no mortality observed in any of our patients sample below 45 years. Our study showed that more patients that suffered HS died when compared to those who suffered IS type of stroke and this is consistent with findings reported by other studies in Africa 9., 13.. The high mortality rate observed within the first one week of hospital admission may be directly due to the effect of neurological damage occasioned by raised intracranial pressure [4]. This is contrary to findings from studies in developed countries who reported significant reductions in stroke mortality. This may be due to their established and well-organized stroke services 1., 7.. More male mortality observed in this study is consistent with the findings of a similar study by Obiako et al. [23]. In contrast, other studies showed that women had poor stroke outcomes than men 13., 24..

In our study, the independent predictors of stroke mortality were old age (>65 years), poor GCS scores (<8), uncontrolled B.P (≥140/90), presence of atrial fibrillation, convulsion, heart failure, and recurrent stroke. Ageing is a non-modifiable risk factor for stroke and old age is linked with several comorbidities, such as cardiovascular disease, malignancies and diabetes 14., 23.. Several studies have reported these diseases as significant causes of stroke mortality in sub-Sahara African (SSA) 9., 23.. Poor GCS score was the most independent predictor of stroke mortality observed in this study. It is noted that more than half of the patients in this study who had low GCS scores died. The impact of the physiological defect was huge. A similar result was noted in previous Nigerian studies 8., 9.. The high mortality observed in people with poor GCS score may be due to raised intracranial pressure and transtentorial herniation [29]. The majority of our patients presented with high blood pressure, which is consistent with other studies' findings 9., 23.. Many studies in LMICs have shown that poor B·P control is linked with poor outcomes, particularly in patients with impaired consciousness 9., 23.. The result of this study has re-emphasized the significance of systemic hypertension as a cause of stroke morbidity and mortality in SSA 9., 23.. Hence, clinicians who manage patients with stroke should recognize the factors that may be responsible for poor B.P control. The understanding of these factors may therefore enhance the reduction in stroke mortality 14., 23.. Furthermore, cardiac diseases like atrial fibrillation and heart failure were associated with stroke mortality in this study. Mortality from atrial fibrillation may be due to occlusion of cerebral arteries, which has been reported by several studies 5., 20.. A study by Isezuo et al. revealed that stroke mortality caused by heart failure is due to systemic hypertension among black Africans [30]. Hence, early detection and control of systemic hypertension therefore should be more aggressively pursued. Repeat stroke, in this study, was associated with stroke mortality. This agrees with the study by Lekoubou et al., which reports that repeat stroke constituted 43% of mortality when compared with first-ever stroke [31]. Therefore, clinicians who manage patients with stroke should recognize the reasons (risk factors) that contributed to the first episode of stroke. This may be necessary in order to provide focussed care to prevent the risk of recurrence.

The study attempted to determining the predictors of stroke mortality that would guide clinicians and other stakeholders who manage patients with stroke on ways of improving outcomes. Though the study centre is located in a rural community, the patients' access to CT imaging and ECG validates the study. As part of the limitations, the study is retrospective, single-centred and hospital-based with a small sample size rather than a prospective, longitudinal community-based study. Hence, it may be subjected to referral bias and might not reflect the community's actual pattern of stroke mortality. Also, the predictors of stroke mortality identified in this study were generalized for stroke, irrespective of subtypes. We may, therefore, have underestimated or overestimated the predictors of mortality for each stroke subtype. Lack of postmortem on the possible cause of death is also a limitation.

## Conclusion

In this study, a total of 276 patients with stroke were studied. The most common clinical presentations were hemiparesis and cranial nerve deficit. The 7-day fatality was 10.1%. The predictors of stroke mortality were poor GCS scores, poor B.P control, presence of atrial fibrillation, heart failure, convulsion and a repeat stroke. The study further strengthens the argument on the high prevalence of stroke, and as a cause of mortality in rural Southwestern Nigeria. Effective communication strategies for primary stroke prevention, early detection, and management of stroke complications may be needed to reduce stroke mortality in rural settings. Our findings may provide an impetus for future prospective research on this outcome.

## Dissemination of results

The results of this study were shared with members of staff of the Emergency Centre through an informal presentation. The results were also published in the service's newsletter.

## Authorship contribution statement

Authors contributed as follow to the conception or design of the work; the acquisition, analysis or interpretation of data for the work; and drafting the work or revising it critically for important intellectual content: AOI contributed 70%; OMS; TAA and PO contributed 10% each. All the authors read and approved the final version of the manuscript to be published and agreed to be accountable for all aspects of the work.

## Declaration of competing interest

The authors declare that they have no conflicts of interest.
